# Association of Shared Living Spaces and COVID-19 in University Students, Wisconsin, USA, 2020

**DOI:** 10.3201/eid2711.211000

**Published:** 2021-11

**Authors:** John Paul Bigouette, Laura Ford, Hannah E. Segaloff, Kimberly Langolf, Juliana Kahrs, Tara Zochert, Jacqueline E. Tate, Douglas Gieryn, Hannah L. Kirking, Ryan P. Westergaard, Marie E. Killerby

**Affiliations:** Centers for Disease Control and Prevention, Atlanta, Georgia, USA (J.P. Bigouette, L. Ford, H.E. Segaloff, J.E. Tate, H.L. Kirking, M.E. Killerby);; Wisconsin Department of Health Services, Madison, Wisconsin, USA (H.E. Segaloff, R.P. Westergaard);; University of Wisconsin–Oshkosh, Oshkosh, Wisconsin, USA (K. Langolf, J. Kahrs, T. Zochert);; Winnebago County Health Department, Oshkosh (D. Gieryn)

**Keywords:** COVID-19, SARS-CoV-2, coronavirus disease 2019, severe acute respiratory syndrome coronavirus 2, viruses, respiratory infections, zoonoses, students, roommates, outbreak, university, dormitory, Wisconsin, United States

## Abstract

We describe characteristics associated with having coronavirus disease (COVID-19) among students residing on a university campus. Of 2,187 students, 528 (24.1%) received a COVID-19 diagnosis during fall semester 2020. Students sharing a bedroom or suite had approximately twice the odds of contracting COVID-19 as those living alone.

In 2020, multiple outbreaks of coronavirus disease (COVID-19), the disease caused by infection with severe acute respiratory syndrome coronavirus 2 (SARS-CoV-2), were documented in institutions of higher education (IHEs; e.g., colleges and universities) across the United States (*1*–*5*). Before students returned to campus, IHEs implemented measures to reduce the spread of SARS-CoV-2 on campus (*6*–*8*). The congregate nature of on-campus residence halls might increase the odds of contracting SARS-CoV-2 because of close-contact exposure, but the association has not been well studied. We describe characteristics of on-campus students associated with having a SARS-CoV-2 infection, including if they shared living spaces, during the fall semester at a Wisconsin, USA, university. 

## The Study

The Centers for Disease Control and Prevention (CDC) partnered with the Wisconsin Division of Health Services (WDHS; Madison, WI, USA) and University of Wisconsin—Oshkosh to investigate COVID-19 among on-campus residents during the fall 2020 semester (September 2–December 19). On-campus residents were housed in 8 dormitories (dorms A–H). In 7 dormitories, students resided in double-occupancy rooms (dorm C included 4 triple-occupancy rooms) and shared bathrooms along with a common area per floor. Dorm D was the only dormitory made up of suites in which <4 students lived in either 4 single-occupancy or 2 double-occupancy bedrooms with the suite’s own bathroom, common area, and kitchen. Not all bedrooms were occupied at their full capacity. After a positive COVID-19 diagnosis, on-campus residents were housed in an isolation dormitory. Students who might have been exposed were housed in a separate quarantine dormitory (*9*,*10*). (Appendix).

Data provided by the university included the number of available rooms, dormitory room types, student housing contracts, serial testing records, and a list of all student COVID-19 cases. For our study, we defined students sharing a bedroom with another student at the start of the semester as having a shared bedroom. Students sharing a suite or defined as having a shared bedroom were classified as having a shared living space. In addition, we defined dormitory floor-level occupancy as the number of occupied rooms divided by the number of rooms per floor. We defined a laboratory-confirmed case as a positive SARS-CoV-2 antigen or reverse transcription PCR test result for any on-campus student during the fall semester (*11*).

All data were analyzed using R version 4.0.2 (R Foundation for Statistical Computing, https://www.r-project.org). We used χ^2^ tests, Fisher exact tests, and t-tests to determine differences between COVID-19 cases and noncases. We modeled the association between student characteristics and a laboratory-confirmed COVID-19 case using univariable and multivariable logistic regression; covariates were age, sex, race, ethnicity, all dormitories, and dormitory floor level occupancy. The dormitory with the lowest COVID-19 positivity for the semester was the reference group. Sharing a bedroom or living space were analyzed in separate models. We conducted our investigation consistent with applicable federal laws and CDC policy (e.g., 45 C.F.R. part 46.102(l)(2), 21 C.F.R. part 56; 42 U.S.C. §241(d); 5 U.S.C. 145 §552a; 44 U.S.C. §3501 et seq.). CDC and WDHS reviewed the investigation; in addition, the university’s ethics review board determined the activities to be nonresearch public health surveillance.

At the start of the fall semester, 2,187 students had on-campus housing contracts. The median age of on-campus students was 19 years; 60.5% were female, 79.4% White, and 79.7% non-Hispanic/Latino (Table 1). Dormitory student populations range was 176–405 students per dormitory, with a mean of 55 students per occupied dormitory floor and a mean floor occupancy of 85% (Table 2) at semester start. Overall, 74.5% of students shared a bedroom and 81.7% of students shared a living space.

**Table 1:** Demographics of on-campus university students in study of coronavirus disease transmission, Wisconsin, USA, September 2–December 19, 2020

Characteristic	Overall		COVID-19 cases*	Non–COVID-19 cases	p value
Total no. persons	2,187		528	1,659	
Age, y, mean (SD)	19.3 (1.1)		19.3 (1.2)	19.2 (0.9)	<0.001
Sex, no. (%)					
F	1,326 (60.6)		324 (61.3)	1,002 (60.4)	0.641
M	820 (37.5)		192 (36.4)	628 (37.8)	
Unknown	41 (1.9)		12 (2.3)	29 (1.8)	
Race, no. (%)					0.017
Alaska Native or Native American	13 (0.6)		5 (0.9)	8 (0.5)	
Asian	86 (3.9)		13 (2.5)	73 (4.4)	
Black or African American	99 (4.5)		20 (3.8)	79 (4.8)	
Native Hawaiian or other Pacific Islander	33 (1.5)		1 (0.2)	32 (1.9)	
White	1,737 (79.4)		434 (82.2)	1,303 (78.5)	
Other	23 (1.1)		2 (0.4)	21 (1.3)	
Unknown/declined	196 (9.0)		53 (10.0)	143 (8.6)	
Ethnicity, no. (%)					0.014
Hispanic or Latino	127 (5.8)		17 (3.2)	110 (6.6)	
Not Hispanic or Latino	1,744 (79.7)		431 (81.6)	1,313 (79.2)	
Unknown/declined	316 (14.5)		80 (15.2)	236 (14.2)	
Dormitory, no. (%)					<0.001
Dorm A	176 (8.1)		33 (6.2)	143 (8.6)	
Dorm B†	206 (9.4)		40 (7.6)	166 (10.0)	
Dorm C	313 (14.3)		78 (14.8)	235 (14.2)	
Dorm D‡	269 (12.3)		83 (15.7)	186 (11.2)	
Dorm E	264 (12.1)		45 (8.5)	219 (13.2)	
Dorm F†	405 (18.5)		126 (23.9)	279 (16.8)	
Dorm G†	204 (9.3)		53 (10.0)	151 (9.1)	
Dorm H	350 (16.0)		70 (13.3)	280 (16.9)	
Shared bedroom, no. (%)§					0.001
Yes	1,630 (74.5)		423 (80.1)	1,207 (72.8)	
No	557 (25.5)		105 (19.9)	452 (27.2)	
Shared living space, no. (%)†					<0.001
Yes	1,787 (81.7)		472 (89.4)	1,315 (79.3)	
No	400 (18.3)		56 (10.6)	344 (20.7)	

*A laboratory-confirmed case was defined as a positive SARS-CoV-2 antigen or reverse transcription PCR test result for any on-campus student during the fall semester. †First-year student dormitories. ‡Only suite-style dormitory made up of suites where <4 students were housed in either 4 single-occupancy or 2 double-occupancy bedrooms with the suite’s own bathroom, common area, and kitchen. §Students who share a bedroom with >1 students.

**Table 2:** Characteristics of living situations for on-campus students at a university in study of coronavirus disease transmission, Wisconsin, USA, September 2–December 19, 2020

Characteristic	Dormitory*								
	A	B†	C	D‡	E	F†	G†	H	Overall
Suite-style dormitory‡	No	No	No	Yes	No	No	No	No	NA
No. occupied floors	4	4	4	5	7	8	4	4	40
No. rooms	122	120	234	264	259	240	115	253	1,607
No. occupied bedrooms	107	110	202	216	186	218	108	223	1,307
Overall dormitory occupancy rate, %	87.7	91.7	86.3	81.8	71.8	90.8	93.9	88.1	81.3
Dormitory floor occupancy rate,§ mean % (SD)	87.7 (0.5)	90.1 (15.9)	86.3 (6.0)	82.1 (6.7)	72.2 (14.9)	86.8 (9.3)	89.9 (7.7)	88.1 (2.9)	85.4 (8.0)
No. student population	176	206	313	269	264	405	204	350	2,187
Students per dormitory floor, mean (SD)	44.0 (6.6)	51.5 (15.2)	78.3 (14.1)	53.8 (5.9)	37.7 (10.6)	50.6 (12.0)	51.0 (12.7)	87.5 (14.8)	54.7 (11.5)
No. COVID-19 cases¶	33	40	78	83	45	126	53	70	528
% Students positive	18.8	19.4	24.9	30.9	17.0	31.1	26.0	20.0	24.1

*Each dormitory floor had a shared common space and bathrooms except dorm D. †First-year student dormitories. ‡Only suite-style dormitory comprised of suites where <4 students were housed in either 4 single- or 2 double-occupancy bedrooms with the suite’s own bathroom, common area, and kitchen. §Dormitory floor-level occupancy was defined as the number of occupied rooms divided by the number of rooms per floor. ¶A laboratory-confirmed case was defined as a positive severe acute respiratory syndrome coronavirus 2 antigen or reverse transcription PCR test result for any on-campus student during the fall semester.

**Figure 1:**
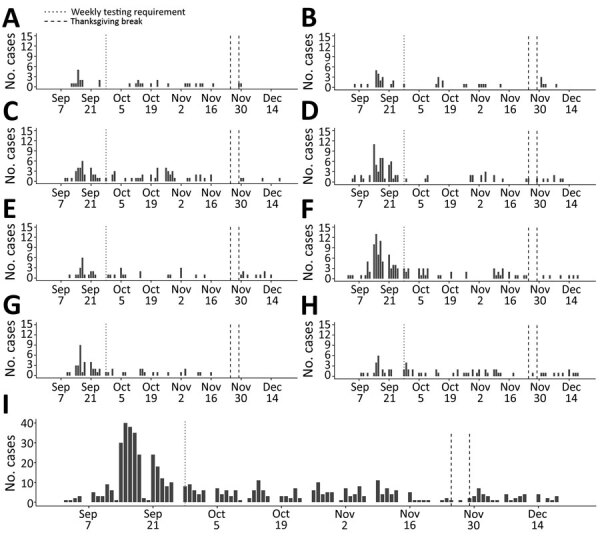
Epidemic curves of daily coronavirus disease cases in each of 8 dormitories (A–H) and overall (I) for a total of 528 cases at a university in Wisconsin, USA, September 2–December 19, 2020. Vertical dotted lines indicate the change in testing requirement from biweekly to weekly. On-campus students returning after the Thanksgiving break (November 25–29, 2020; vertical dashed lines) were required to test before leaving campus and twice >48 hours apart upon returning to campus. Dorms A, B, F, and G house first-year students. Dorm D is made up of suites of 4 single- or 2 double-occupancy bedrooms with a shared bathroom, common area, and kitchen.

During the semester, 528 (24.1%) COVID-19 cases were identified among on-campus students. The percentage of students diagnosed with COVID-19 was 17.0%–31.1% across dormitories for the fall semester; the lowest percent positivity was in dorm E. All dormitories saw a rise in cases in mid- to late September (Figure 1).

**Figure 2:**
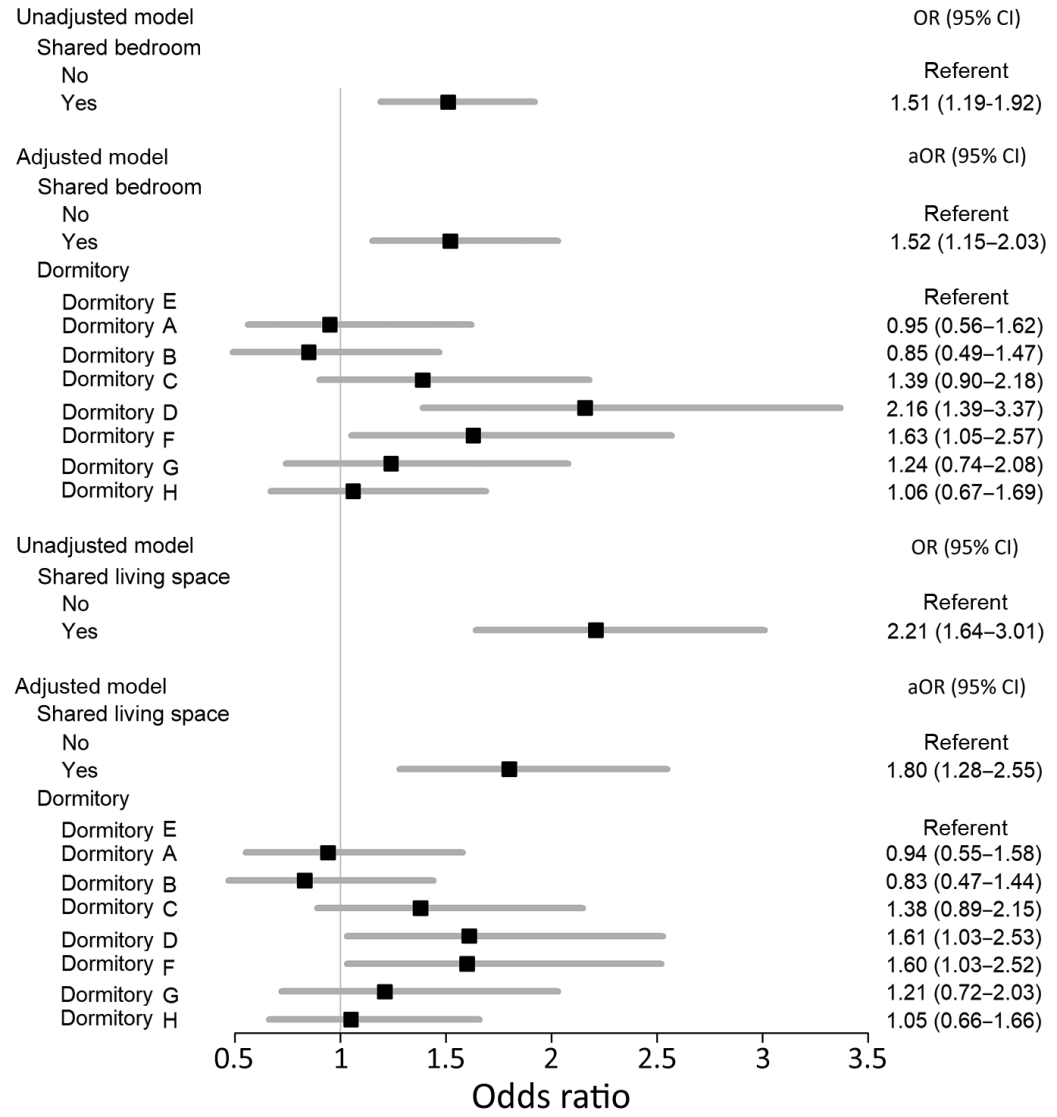
Associations between shared living spaces and coronavirus disease (COVID-19) at a university (N = 2,187), Wisconsin, USA, September 2–December 19, 2020. Black boxes indicate odds ratios; gray bars indicate 95% CIs. Models were adjusted for age, sex, race, ethnicity, dormitories, and floor level occupancy (%). Shared living space was defined as one in which >2 students share either a bedroom or suite. Dorms A, B, F, and G housed first-year students. Dorm E was selected as the reference group because it had the lowest semester COVID-19 positivity among on-campus residents. Dorm D is composed of suites of 4 single-occupancy or 2 double-occupancy bedrooms with a shared bathroom, common area, and kitchen. aOR, adjusted odds ratio; OR, odds ratio.

Using a univariable regression model, we found that students who shared a bedroom had 1.52 (95% CI 1.19–1.92) times the odds of receiving a COVID-19 diagnosis as students who lived alone (Figure 2). The effect estimate remained unchanged in the adjusted multivariable regression model (Appendix Table 1). However, in the adjusted model, students with a shared living space (e.g., suites and bedrooms) had 1.80 (95% CI 1.28–2.55) times the odds of testing positive for COVID-19 compared with students living alone. After controlling for shared living status, students from 2 dormitories, dorms D and F, had higher odds for COVID-19 than dorm E students in both models.

CDC has provided guidance on prevention measures to reduce the transmission of SARS-CoV-2 at IHEs (*6*,*8*). Similar to other IHE outbreak reports from the United States, the university saw a surge in cases during September (*1*,*3*–*5*). After the surge, the university updated their COVID-19 prevention plan such that residential students were tested weekly instead of biweekly for SARS-CoV-2, messaging on COVID-19 prevention measures increased, and on-campus dining was limited to takeout only for 2 weeks.

## Conclusions

Despite this university’s updated COVID-19 prevention plan, students sharing a suite or bedroom had higher odds of being diagnosed with COVID-19. SARS-CoV-2 household transmission studies have shown that households are a significant transmission source for both symptomatic and asymptomatic persons (*12*,*13*). For example, a meta-analysis found that the household SARS-CoV-2 secondary attack rate was 16.6% (*8*,*12*). Reducing the number of students with roommates or those in suite-style units is needed to limit SARS-CoV-2 transmission.

After adjusting for sharing bedrooms or living spaces, students from 2 dormitories still had higher odds of having COVID-19 than students from the dormitory with the lowest percentage of positive students. This finding could be associated with differing student attitudes and social behaviors towards COVID-19 (*14*).

Racial and ethnic disparities in COVID-19 incidence have been found in persons <25 years of age in the United States (*15*). However, we found that Native Hawaiian or other Pacific Islander students had lower odds of having COVID-19 compared with White students, and Hispanic students had lower odds than non-Hispanic students: adjusted odds ratio for Native Hawaiian or other Pacific Islander students was 0.13 (95% CI 0.01–0.63) and for Hispanic students it was 0.56 (95% CI 0.31–0.96). We observed no other associations by age, sex, race category, and dormitory floor occupancy. These results should be interpreted with caution; our findings could be the result of low sample sizes in some groups or residual confounding.

The first limitation of our study is that findings from this IHE may not be generalizable for all IHEs. Second, these results characterize an association between sharing a living space and COVID-19 and do not necessarily indicate roommate transmission. Third, students may have moved out of the dormitory during the semester, causing an underestimation of attack rates and misclassification of those students with roommates or suitemates for the term. Last, because this investigation was cross-sectional in design, a causal relationship cannot be determined.

In summary, sharing a living space or bedroom was associated with increased odds of having COVID-19 even with COVID-19 prevention policies at a Wisconsin university. Reducing the number of students sharing living spaces could further prevent the spread of SARS-CoV-2 on-campus as part of COVID-19 prevention practices at IHEs.

Appendix Additional information about the association between shared living spaces and coronavirus disease at University of Wisconsin, Oshkosh, Wisconsin, USA, September–December 2020.
