# Supplementary material for: Association of Shared Living Spaces and COVID-19 in University Students, Wisconsin, USA, 2020
Source: Emerg Infect Dis. 2021 Nov;27(11):2882–6. doi: 10.3201/eid2711.211000 (PMC8544959; doi:10.3201/eid2711.211000)
Supplement: Appendix — Additional information about the association between shared living spaces and coronavirus disease at University of Wisconsin, Oshkosh, Wisconsin, USA, September–December 2020. [file 21-1000-Techapp-s1.pdf]

Article DOI: https://doi.org/10.3201/eid2711.211000

Association of Shared Living Spaces and COVID-19 in University Students, Wisconsin, USA, 2020

Appendix

Summary of COVID-19 Mitigation Practices

At the university, all students signed a pledge at the beginning of the semester to wear masks when not sleeping, bathing, or eating; practice physical distancing; not congregate in groups; and partake in free serial coronavirus disease 2019 (COVID-19) testing biweekly. Course instruction was delivered in a mix of in-person, online, and hybrid classes. After a rise in COVID-19 cases on campus, the university updated their COVID-19 mitigation strategy at the end of September. On September 28, 2020, on-campus students were required to be tested weekly for COVID-19. In addition, all dining hall services were moved to take-out only for 2 weeks. For Thanksgiving break (November 25–29, 2020), on-campus students returning home were asked to test before leaving campus and twice at least 48 hours apart the week they returned to the university. Students did not have to complete the testing requirement following Thanksgiving break prior to returning to in-person classes. COVID-19 positive students were granted a 90-day exemption window from the time of their positive diagnosis and had to begin enrolling in serial COVID-19 following this period.

Contact Tracing

Students who tested positive for COVID-19 outside of the university's testing center were asked to self-report their case through an online reporting form to notify the university. Students were asked to list the names of anyone they had close contact with while on campus. Names were shared with local county health officials for contact tracing. A school disease investigator/contact tracer was assigned to all on-campus student cases. Isolation housing was designated for individuals with positive cases or others requiring isolation based on referral from

a public health authority or healthcare provider. Quarantine housing (14 days) was provided for any on-campus student with a close physical association (i.e., roommate) to a person with a positive coronavirus test or who was referred by a public health authority or Student Health Center.

Appendix Table. Logistic regression results in study of association between shared living spaces and coronavirus disease 2019 at a university, Wisconsin, United States, September 2–December 19, 2020*

Variable	Unadjusted models OR (95% CI)	Shared bedroom adjusted model† aOR (95% CI)	Shared living space adjusted model† aOR (95% CI)
Shared bedroom‡			
No	Reference	Reference	NA
Yes	**1.51 (1.19–1.92)**	**1.52 (1.15–2.03)**	NA
Shared living space§			
No	Reference	NA	Reference
Yes	**2.21 (1.64–3.01)**	NA	**1.80 (1.28–2.55)**
Age, y	**0.86 (0.78–0.94)**	0.92 (0.82–1.04)	0.95 (0.84–1.07)
Sex			
F	Reference	Reference	Reference
M	0.95 (0.77–1.16)	1.09 (0.88–1.36)	1.09 (0.88–1.35)
Unknown	1.28 (0.62–2.47)	1.12 (0.51–2.39)	1.11 (0.50–2.37)
Race			
White	Reference	Reference	Reference
Alaska Native or Native American	1.88 (0.56–5.65)	2.10 (0.63–6.48)	2.00 (0.59–6.17)
Asian	**0.53 (0.28–0.94)**	0.63 (0.33–1.12)	0.63 (0.33–1.12)
Black or African American	0.76 (0.44–1.23)	0.90 (0.52–1.48)	0.90 (0.53–1.48)
Native Hawaiian or other Pacific Islander	**0.09 (0.01–0.44)**	**0.13 (0.01–0.61)**	**0.13 (0.01–0.63)**
Other	0.29 (0.05–0.98)	0.45(0.07–1.70)	0.44 (0.07–1.62)
Unknown/declined	1.11 (0.79–1.53)	1.07 (0.62–1.89)	1.07 (0.62–1.89)
Ethnicity			
Not Hispanic or Latino	Reference	Reference	Reference
Hispanic or Latino	**0.47 (0.27–0.77)**	**0.56 (0.31–0.94)**	**0.56 (0.31–0.96)**
Unknown/declined	1.03 (0.78–1.36)	1.01 (0.64–1.57)	1.01 (0.64–1.57)
Dormitory			
Dorm E	Reference	Reference	Reference
Dorm A	1.12 (0.68–1.84)	0.95 (0.56–1.62)	0.94 (0.55–1.58)
Dorm B¶	1.17 (0.73–1.88)	0.85 (0.49–1.47)	0.83 (0.47–1.44)
Dorm C	1.62 (1.07–1.87)	1.39 (0.90–2.18)	1.38 (0.89–2.15)
Dorm D#	**2.17 (1.44–3.30)**	**2.16 (1.39–3.37)**	**1.61 (1.03–2.53)**
Dorm F¶	**2.20 (1.51–3.25)**	**1.63 (1.05–2.57)**	**1.60 (1.03–2.52)**
Dorm G¶	1.71 (1.09–2.68)	1.24 (0.74–2.08)	1.21 (0.72–2.03)
Dorm H	1.22 (0.81–1.85)	1.06 (0.67–1.69)	1.05 (0.66–1.66)
Floor-level room occupancy, (%)**	1.00 (0.99–1.02)	1.00 (0.99–1.02)	1.00 (0.99–1.02)

*N = 2,187. Bold text indicates p<0.05.aOR, adjusted odds ratio; NA, not applicable; OR, odds ratio.
†Full model adjusted for age, sex, race, ethnicity, dormitories, and floor level occupancy (%)
‡Students who share a bedroom with $\geq$1 students.
§Students who share either a dormitory bedroom or suite (e.g., suite-style dormitory comprised of suites where $\leq$4 students were housed in either 4 single- or 2 double-occupancy bedrooms with the suite's own bathroom, common area, and kitchen) with one or more students.
¶First-year student dormitories.
#Dormitory composed of suite-style units with their own common area, bathroom, and kitchen.
**Dormitory floor-level occupancy was defined as the number of occupied rooms divided by the number of rooms per floor.
